# Regulation of Macropinocytosis by Diacylglycerol Kinase ζ

**DOI:** 10.1371/journal.pone.0144942

**Published:** 2015-12-23

**Authors:** Ryan Ard, Kirk Mulatz, Julia L. Pomoransky, Robin J. Parks, Laura Trinkle-Mulcahy, John C. Bell, Stephen H. Gee

**Affiliations:** 1 Department of Cellular and Molecular Medicine, University of Ottawa, 451 Smyth Rd, Ottawa, ON, K1H 8M5, Canada; 2 Centre for Neuromuscular Disease, University of Ottawa, Ottawa, ON, Canada; 3 Centre for Innovative Cancer Research, Ottawa Hospital Research Institute, 503 Smyth Road, 3rd floor ORCC, Ottawa, ON, K1H 1C4, Canada; 4 Department of Biochemistry, Microbiology and Immunology, University of Ottawa, Ottawa, ON, Canada; 5 Regenerative Medicine Program, Ottawa Hospital Research Institute, 501 Smyth Road, Ottawa, ON, K1H 8L6, Canada; 6 Ottawa Institute of Systems Biology, University of Ottawa, Ottawa, ON, Canada; University of Toledo, UNITED STATES

## Abstract

Macropinosomes arise from the closure of plasma membrane ruffles to bring about the non-selective uptake of nutrients and solutes into cells. The morphological changes underlying ruffle formation and macropinosome biogenesis are driven by actin cytoskeleton rearrangements under the control of the Rho GTPase Rac1. We showed previously that Rac1 is activated by diacylglycerol kinase ζ (DGKζ), which phosphorylates diacylglycerol to yield phosphatidic acid. Here, we show DGKζ is required for optimal macropinocytosis induced by growth factor stimulation of mouse embryonic fibroblasts. Time-lapse imaging of live cells and quantitative analysis revealed DGKζ was associated with membrane ruffles and nascent macropinosomes. Macropinocytosis was attenuated in DGKζ-null cells, as determined by live imaging and vaccinia virus uptake experiments. Moreover, macropinosomes that did form in DGKζ-null cells were smaller than those found in wild type cells. Rescue of this defect required DGKζ catalytic activity, consistent with it also being required for Rac1 activation. A constitutively membrane bound DGKζ mutant substantially increased the size of macropinosomes and potentiated the effect of a constitutively active Rac1 mutant on macropinocytosis. Collectively, our results suggest DGKζ functions in concert with Rac1 to regulate macropinocytosis.

## Introduction

Macropinocytosis is a form of endocytosis in which extracellular fluid and plasma membrane are internalized in large vesicles [1,2]. Macrophages and immature dendritic cells use constitutive macropinocytosis to sample molecules for antigen presentation [3,4]. In most cells however, it is a transient response to growth factor stimulation and represents an efficient route for the non-selective uptake of nutrients and solutes. Macropinocytosis also contributes to infection since many pathogenic bacteria and viruses exploit it as a pathway to gain entry into cells [5–7]. It has also been implicated in the modulation of cell-cell adhesion by regulating the internalization of E-cadherin-catenin complexes [8,9]. Moreover, recent evidence suggests Ras-transformed cancer cells use macropinocytosis to internalize extracellular protein to supply amino acids for their proliferation and growth [10]. Thus, macropinocytosis is a fundamental cellular process used by a variety of cell types and underpins several different biological functions.

Macropinosomes are large diameter (0.2–5 um) vesicles formed from actin-rich, sheet-like extensions of the plasma membrane called ruffles [1]. Most ruffles dissolve back into the plasma membrane, but some peripheral ruffles fold back on themselves, forming fluid-filled compartments. Other, circular-shaped ruffles form open, cup-like membrane extensions that close and trap extracellular fluid and solutes [2]. In both cases, macropinosome formation requires constriction of the distal margin of these pockets followed by membrane fusion and fission events to separate the macropinosome from the plasma membrane [1]. Newly formed macropinosomes then transition into early endosomes and eventually fuse with lysosomes [11].

Organized movements of membranes and the actin cytoskeleton are coordinated during membrane ruffling and macropinocytosis by a variety of signaling molecules. Membrane phosphoinositides, protein and lipid kinases, and Rho GTPases have all been implicated [2]. Generally speaking, discrete changes in phospholipid species in ruffles and macropinocytic cups at various stages of their formation induce coordinated changes in the activities of molecules that regulate actin organization, particularly Rho GTPases [1,12–14].

Rho GTPases are key regulators of actin organization. They function like molecular switches, cycling between inactive GDP-bound and active GTP-bound states [15]. In their GTP-loaded conformation they bind downstream effectors to elicit actin reorganization. Expression of a GTPase-defective, and thus constitutively active, mutant of the Rho GTPase Rac1 induces extensive membrane ruffling and macropinocytosis in fibroblasts [16] and plays a role in the transformation of membrane ruffles into macropinosomes [17]. GTP-bound Rac1 binds to and activates a variety of effectors that regulate lamellipodia formation and membrane ruffling including p21-activated kinase 1 (PAK1) [18,19]. Activated PAK1 stimulates macropinocytosis [20] and the PAK1 target ctBP1/BARS is required for scission of the macropinocytic cup from the plasma membrane [21]. Thus, proper regulation of Rac1 activity is important for driving the cellular changes required for membrane ruffling and macropinocytosis.

Diacylglycerol kinases (DGKs) are enzymes that phosphorylate diacylglycerol (DAG) to yield phosphatidic acid (PA). The ten mammalian DGK isozymes differ in structure, patterns of expression, enzymatic properties and subcellular localization, suggesting they modify distinct DAG signaling events and are regulated by separate molecular mechanisms [22,23]. By metabolizing DAG, DGKs simultaneously attenuate the activity of DAG-activated proteins and stimulate the activity of proteins activated by PA [24]. Previously, we showed the zeta isoform (DGKζ) acts upstream of Rac1 and contributes to its activation by releasing it from its inhibitor RhoGDI [25]. DGKζ exists in a multi-protein signaling complex with Rac1, PAK1 and RhoGDI and the scaffold protein syntrophin. DGKζ-derived PA activates PAK1, which then phosphorylates RhoGDI to trigger Rac1 release [25,26]. Collectively, our findings established a mechanism whereby a change in the lipid second messenger PA modulates the amount of active Rac1 and thus contributes to the regulation of the actin cytoskeleton.

Here, we demonstrate that growth factor-induced macropinocytosis is defective in fibroblasts lacking DGKζ. We show DGKζ is associated with membrane ruffles and nascent macropinosomes. In addition, we provide evidence that DGKζ, in concert with Rac1, regulates the size of macropinosomes. Finally, our analyses reveal that DGKζ is required for ruffling and macropinocytosis induced by constitutively active Rac1. Together, these results suggest a novel role for DGKζ in linking lipid signaling to Rac1-dependent macropinosome biogenesis.

## Results

### Defective macropinocytosis in DGKζ-null cells

**Fig 1:**
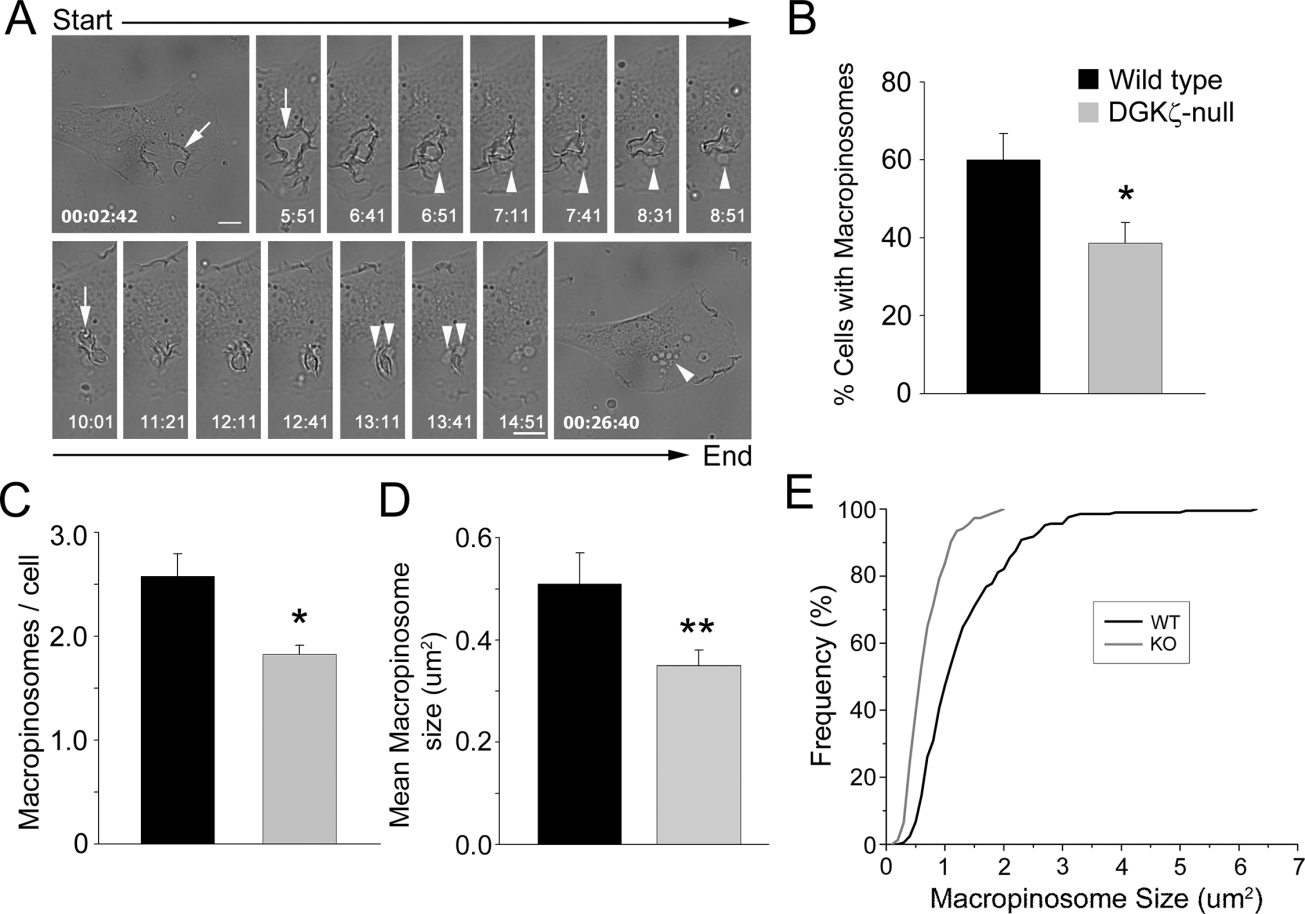
Quantitative analysis of macropinocytosis in living wild type and DGKζ-null fibroblasts. (A) Representative phase-contrast images captured from time-lapse video microscopy of PDGF-stimulated, wild type fibroblasts show macropinosomes (arrowheads) forming from membrane ruffles (white arrows). Two separate sequences (top and bottom) are shown from a 30 min video. Scale bar, 10 μm. (B-D) Graphs showing the percentage of cells with macropinosomes (B), the number of macropinosomes per cell (C) and the mean macropinosome size (D) in wild type and DGKζ-null fibroblasts following 30 min PDGF stimulation. Values are the mean ± SEM from at least four independent experiments. A single asterisk denotes a significant difference (p<0.05) and two asterisks, a highly significant difference (p<0.01), between wild type and null cells by Student’s t test. (E) Cumulative frequency distribution showing the distribution of macropinosome sizes (defined as the two-dimensional area) in PDGF-stimulated wild type and null cells.

We showed previously that membrane ruffling, a prerequisite for macropinocytosis, is deficient in DGKζ-null cells [25]. To investigate potential roles for DGKζ in macropinocytosis, we analyzed phase contrast, time-lapse images of nascent macropinosomes forming from membrane ruffles following stimulation with platelet-derived growth factor (PDGF; S1, S2 and S3 Movies). To minimize the possibility that potential differences in macropinocytosis between wild type and DGKζ-null cells could be due to defects in the process of membrane ruffle formation per se, we only scored macropinosomes that were clearly derived from already-formed membrane ruffles (see Materials and Methods; Fig 1A). In this way, we could distinguish membrane ruffling defects from possible defects in macropinosome formation. In other words, we measured successful transitions from membrane ruffles to macropinosomes. If a membrane ruffle fails to give rise to a macropinosome, then we can conclude that the failure occurred at a point downstream of ruffle formation.

Following stimulation with PDGF, wild type cells were significantly more likely to form macropinosomes from ruffles (Fig 1B) and had, on average, more macropinosomes per cell than DGKζ-null cells (Fig 1C). Moreover, the mean macropinosome size in wild type cells was significantly larger than in null cells (Fig 1D). A cumulative frequency distribution showed that macropinosomes in null cells did not have areas larger than ~2 um^2^, whereas those in wild type cells often exceeded 2 um^2^ (Fig 1E). These observations suggest a defect in PDGF-induced macropinosome formation in fibroblasts lacking DGKζ.

**Fig 2:**
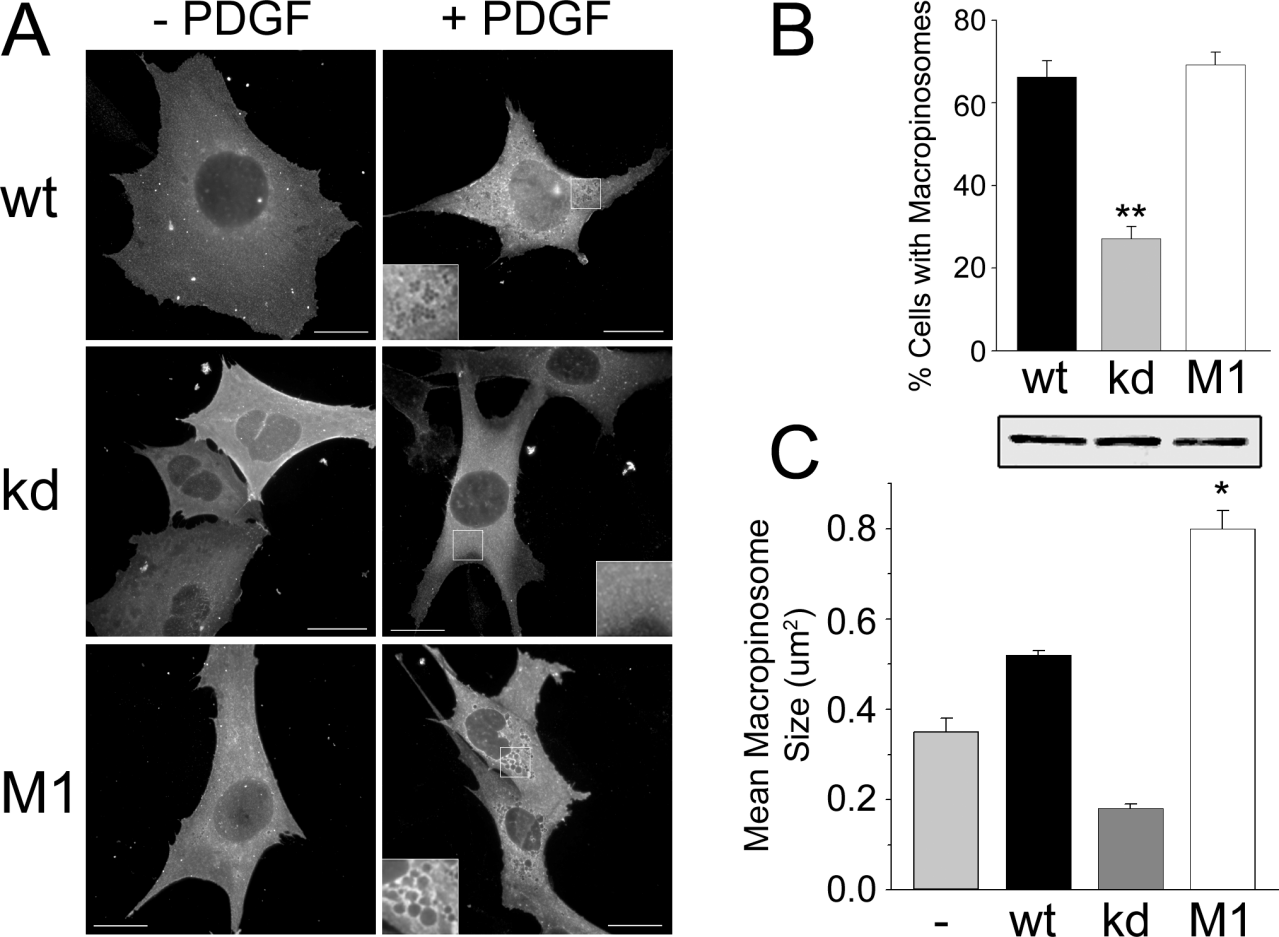
Rescue of PDGF-induced macropinocytosis. DGKζ-null cells were infected with adenoviruses bearing HA-tagged versions of wild type (wt), kinase dead (kd), or phosphomimetic (M1) DGKζ. (A) Representative images of infected cells with (+ PDGF) and without (- PDGF) stimulation. Insets show magnified images of the regions indicated by the white boxes. Scale bars, 20 um. (B—C) Quantification of macropinocytosis in infected DGKζ-null cells. The graphs show the percentage of cells with macropinosomes (B) and the mean macropinosome size (C). Values are the mean ± SEM from three independent experiments. Asterisks denote a significant difference (p<0.05) from kd as determined by a one-way analysis of variance, followed by a Tukey post hoc multiple-comparison test. The western blot shows equivalent levels of HA-DGKζ expression in lysates of infected cells. The lanes correspond to the bars in the graphs above and below.

To demonstrate that the defect in macropinocytosis is due directly to DGKζ loss, a hemagglutinin (HA)-tagged version of the wild type protein was reintroduced into null cells by adenoviral infection. Expression of HA-DGKζ restored PDGF-induced macropinocytosis to wild type levels (Fig 2A and 2B). In contrast, macropinosomes detected in cells expressing a kinase dead mutant (DGKζ^kd^) were significantly smaller and less numerous (Fig 2A–2C). We also tested a mutant (DGKζ^M1^) that mimics serine phosphorylation of the DGKζ MARCKS domain [27]. Compared to the wild type protein, DGKζ^M1^ exhibits increased plasma membrane localization and has greater biological activity in assays of neurite outgrowth [28,29]. Equivalent levels of expression of HA-DGKζ^M1^ in null cells yielded approximately the same percentage of cells containing macropinosomes as wild type DGKζ (Fig 2B), but the macropinosomes produced were substantially larger (Fig 2A–2C). Taken together, these results indicate that the loss of DGKζ enzymatic activity is the primary cause of the macropinocytosis defect in DGKζ-null fibroblasts.

### Decreased uptake of vaccinia virus in DGKζ-null fibroblasts

The observed decrease in macropinocytosis in DGKζ-null cells might be explained by a reduced number of macropinosomes or by a reduction in macropinosome size to below the limit of resolution, in which case the number of macropinosomes might actually be increased, but would not be counted in our assay. Alternatively, both the number and size of macropinosomes might be decreased. To begin to distinguish among these possibilities, we sought to employ an independent method to quantify macropinocytosis in wild type and DGKζ-null cells. The most common method of quantifying macropinocytosis involves the uptake of the soluble enzyme horseradish peroxidase (HRP) from the extracellular environment and measuring enzyme activity in the cell lysates [30]. However, this technique poses limitations including low sensitivity and non-specific binding of HRP to the cell surface. Alternatively, the uptake of fluorescent dextran into cells can be measured using by fluorimetry, but again, the extent to which the dextran adheres non-specifically to cells poses limitations to accurate quantification, especially using cells with low levels of constitutive macropinocytosis [10,21,31].

**Fig 3:**
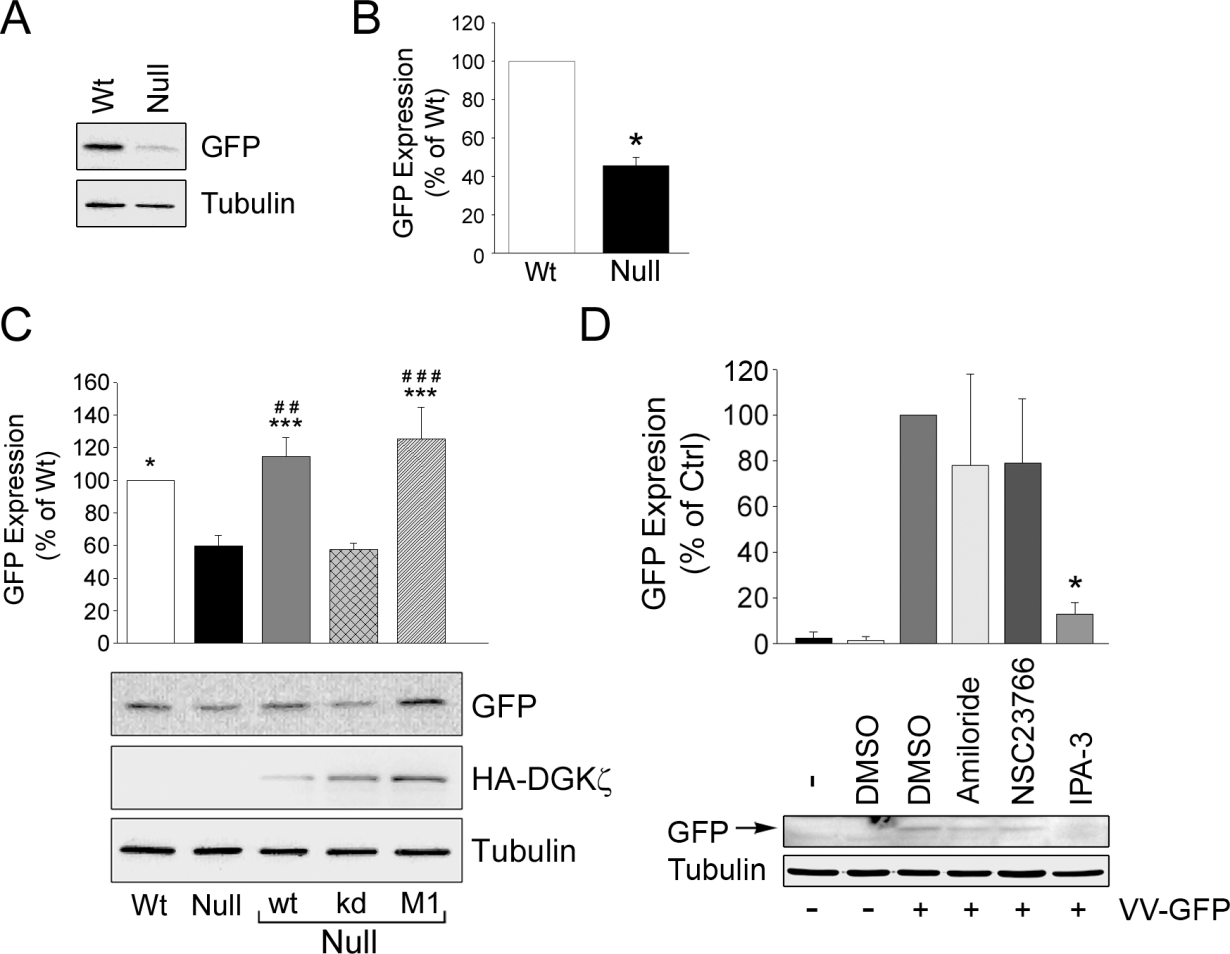
Decreased uptake of Vaccinia virus is an indicator of reduced macropinocytosis. (A) Wild type (Wt) and DGKζ-null (Null) fibroblasts were infected with double-deleted vaccinia virus mature virions engineered to express GFP. GFP expression was assayed by western blotting Tubulin was used as a loading control. (B) Quantification of GFP expression in Wt and Null cells. Values are the mean ± S.E.M. of three independent experiments. The asterisk indicates a statistically significant difference from Wt (p<0.001) by Student’s t-test. (C) Rescue of GFP expression in null cells infected with adenovirus bearing HA-tagged wild type (wt) DGKζ, a kinase-dead mutant (kd), or the MARCKS domain phosphomimetic mutant (M1). GFP expression was assayed by western blotting as in (A) following infection with vaccinia virus. The graph shows the quantification of GFP expression. Statistical analysis was performed by a one-way ANOVA followed by a Holm-Sidak post-hoc multiple comparison test. Asterisks denote a significant difference from uninfected null cells and hashes indicate a significant difference from null cells expressing DGKζ^kd^. * or # = p < 0.05, ** or ## = p < 0.01, *** or ### = p < 0.005. (D) Effect of inhibitors on GFP expression. Wild type fibroblasts infected with VV-GFP were treated either with vehicle alone (DMSO), 100 uM amiloride, 200 uM of the Rac inhibitor NSC 23766, or 40 uM of the PAK1 inhibitor IPA-3. The arrow indicates a GFP band present in lysates of infected, but not uninfected, cells. The graph shows the quantification of western blots from two independent experiments. The asterisk indicates a statistically significant difference (p<0.05) from control (VV-GFP + DMSO).

To circumvent these experimental limitations, we made use of a cellular assay that measures macropinocytosis based on the uptake of vaccinia virus. Mature virions of vaccinia virus (VV) make exclusive use of macropinocytosis to infect mammalian cells [32]. The expression of viral proteins in the infected cells provides a clear quantitative endpoint assay and functional readout of macropinocytosis. This method offers advantages over traditional assays, including greatly improved sensitivity, which is useful when measuring uptake in cells like mouse embryonic fibroblasts (MEFs) that have low levels of constitutive macropinocytosis. To measure differences in macropinocytosis between wild type and DGKζ-null cells, we analyzed the uptake of a VV strain engineered to express GFP in the cytoplasm of infected cells [33]. Importantly, GFP expression is directly proportional to the number of viral genomes in the cell and therefore provides an accurate measure of macropinocytosis. Western blots of VV-infected cells revealed a ~60% decrease in GFP levels in DGKζ-null cells compared to wild type cells (Fig 3A and 3B). Reintroducing HA-DGKζ^wt^ or HA-DGKζ^M1^, but not DGKζ^kd^, into null cells was able to rescue virus uptake as measured by GFP expression (Fig 3C).

Different cell types can internalize VV via several potential mechanisms [34,35], including direct fusion with the plasma membrane [36,37]. To determine the extent that mechanisms other than macropinocytosis contribute to VV-GFP uptake in fibroblasts, we tested several pharmacological inhibitors shown to affect VV uptake by macropinocytosis [32]. In our experiments, amiloride and the Rac1 inhibitor NSC23766 had modest effects on VV-GFP uptake at the concentrations tested, however a selective PAK1 inhibitor potently blocked uptake (Fig 3D). Since PAK1 is essential for macropinocytosis [20] and VV infection [32], these data suggest macropinocytosis is the main route of VV-GFP entry into fibroblasts under these conditions [32]. Collectively, the results from these VV uptake experiments support the proposition that macropinocytosis is deficient in DGKζ-null cells and reaffirm the requirement of DGKζ catalytic activity for macropinocytosis.

**Fig 4:**
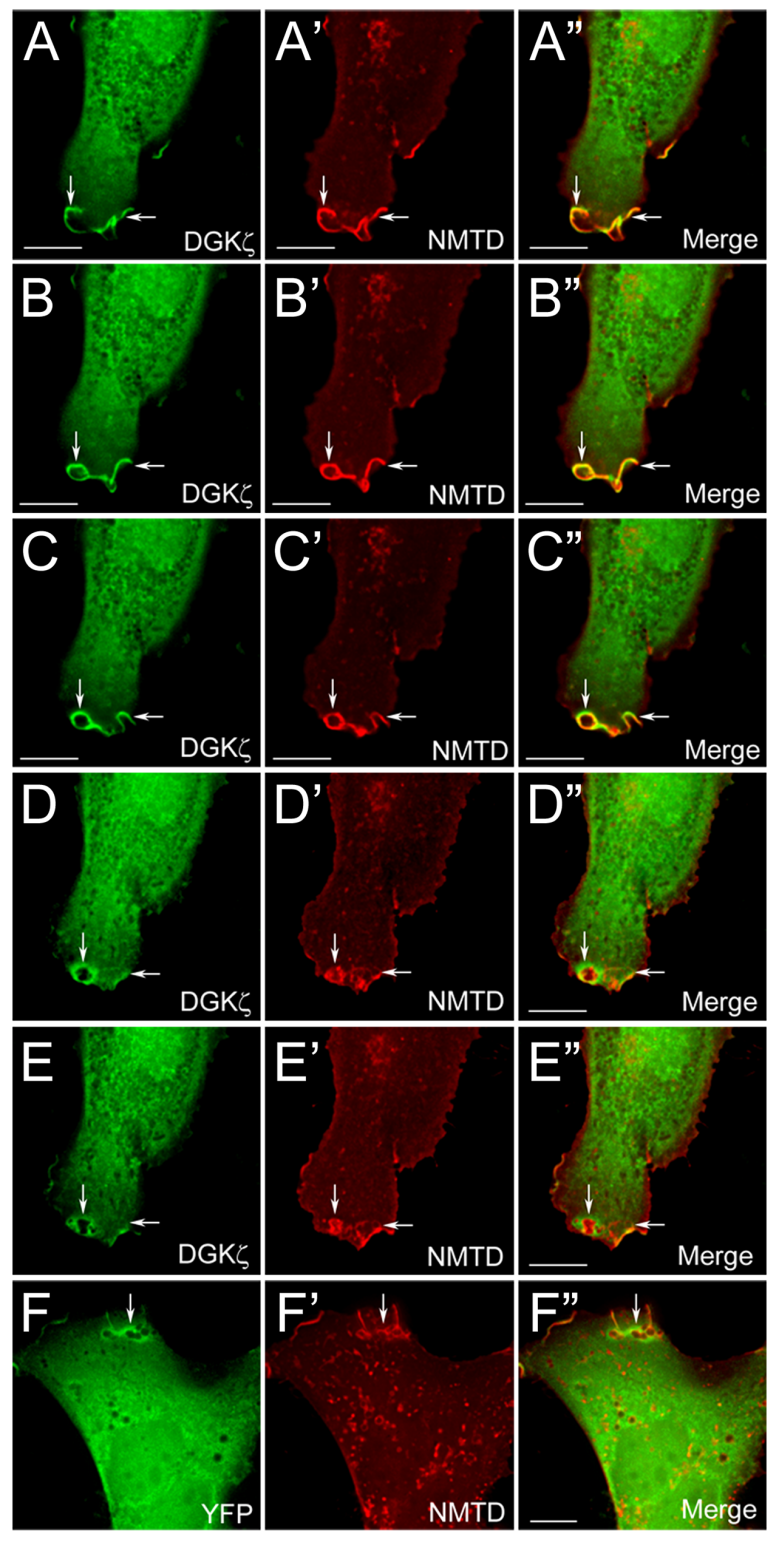
YFP-DGKζ Localization During Macropinosome Biogenesis. Wild type fibroblast cells were cotransfected with plasmids encoding the N-terminal membrane-targeting domain of neuromodulin fused to the N-terminus of mCherry (NMTD-mCherry) and either YFP-DGKζ or YFP alone. Cells stimulated with 50 ηg/ml PDGF were visualized by time-lapse video microscopy. Shown are representative images of YFP-DGKζ (A-E) and NMTD-mCherry (A’-E’) localization in a single optical plane from a time-lapse sequence during various stages of macropinosome biogenesis including: the formation of an irregular ruffle (A and B, horizontal arrows), transition to a curved ruffle (A, vertical arrow and C, horizontal arrow), closure into a circular ruffle or membrane cup (B and C, vertical arrows), and cup closure (D, vertical arrow and E, arrows). Note the high concentration of YFP-DGKζ surrounding the newly formed macropinosomes (D” and E”, vertical arrows). (F-F”) YFP alone also appears to be concentrated around newly formed macropinosomes. Scale bars = 10 um.

To study the role of DGKζ during macropinosome biogenesis, we exploited time-lapse microscopy of living, wild type fibroblasts co-expressing a yellow fluorescent protein (YFP)- DGKζ fusion protein and the N-terminal membrane-targeting domain (20 amino acids) of neuromodulin (GAP-43) fused to the N-terminus of mCherry (NMTD-mCherry). This region of neuromodulin, which contains two cysteine residues that undergo palmitoylation in mammalian cells, is sufficient for plasma membrane and Golgi targeting (Liu et al., 1994). Consistent with this, NMTD-mCherry demarcated membrane ruffles and macropinosome membranes in PDGF-stimulated, wild type MEFs (Fig 4A’–4F’). Three to four minutes after stimulating the cells with PDGF, Z-axis stacks were collected for the green/yellow and red channels every 15–20 seconds from cotransfected cells. YFP-DGKζ appeared to be concentrated in membrane ruffles, in macropinocytic cups, and in newly formed macropinosomes, where it colocalized with NMTD -mCherry (Fig 4A–4C”, arrows and S4 Movie). Additionally, YFP-DGKζ was concentrated in the cytoplasm surrounding newly formed macropinosomes (Fig 4D–4D” and 4E–4E”). However, when we examined cells expressing YFP alone, it also appeared to be concentrated around large, newly formed macropinosomes in lamellipodia (Fig 4F–4F” arrows and S5 Movie), suggesting proteins may non-specifically accumulate there. This likely reflects the fact that many macropinosomes have diameters that are larger than the thickness of the lamellipodium (0.1–0.3 um) and thus distend the plasma membrane, allowing more cytoplasm in that area.

Therefore, to determine if YFP-DGKζ is specifically recruited to the membrane surrounding macropinosomes, we normalized the intensity of YFP (control) or YFP-DGKζ fluorescence signals to the mCherry-NMTD membrane marker, which we reasoned should have constant signal intensity in both conditions. To facilitate comparisons between macropinosomes from different cells and experiments, we restricted our analysis to around the time when macropinosomes first closed. Since time-lapse images were captured at approximately 20 second intervals, the first time point we analyzed was approximately 15–20 seconds after ruffle closure. This was also the time when the YFP-DGKζ signal appeared to be the strongest; when newly formed macropinosomes were changing shape, connecting to and merging with neighboring macropinosomes. The duration of YFP-DGKζ association with macropinosomes was variable but generally decreased during the motile phase when macropinosomes moved centripetally towards the nucleus (Yoshida et al., 2009).

**Fig 5:**
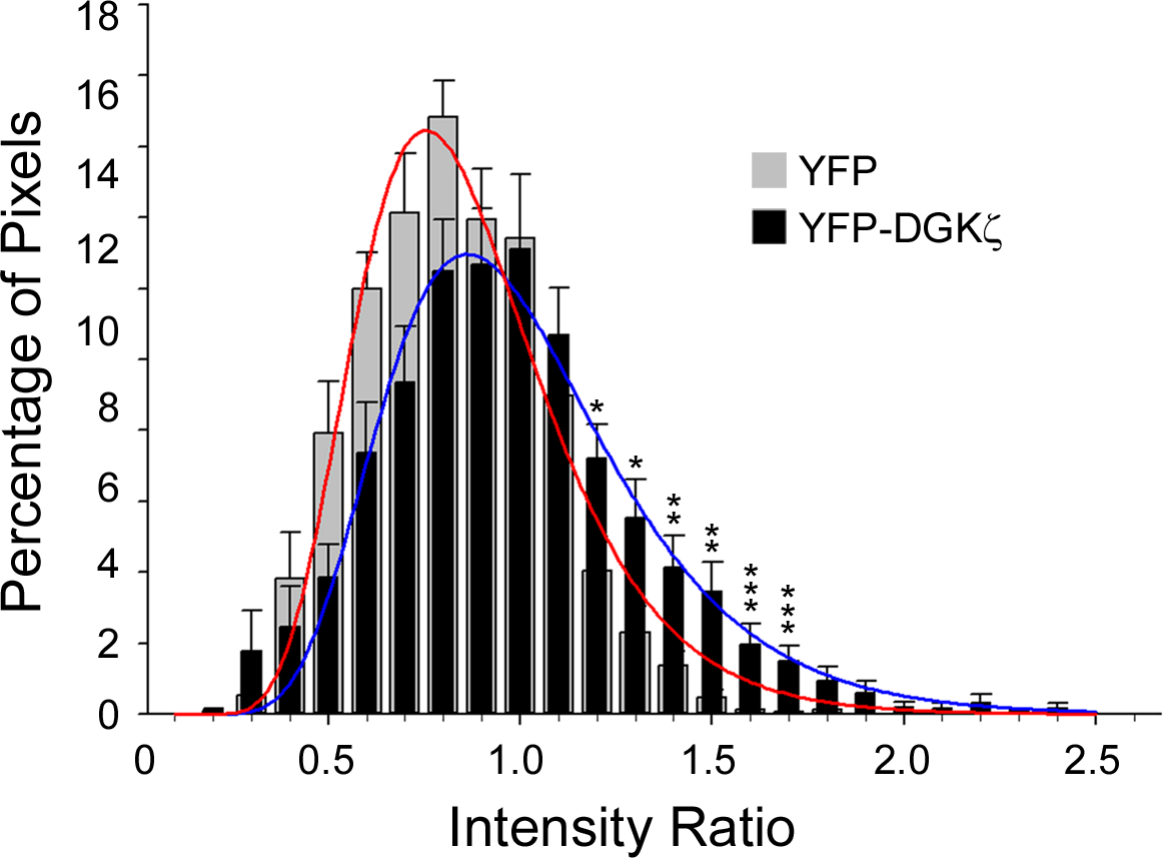
Quantitative analysis of YFP-DGKζ Localization During Macropinocytosis. Ratiometric images of wild type and DGKζ-null cells expressing NMTD-mCherry and either YFP-DGKζ, or YFP alone, taken approximately 15–20 seconds after ruffle closure were analyzed as described in Materials and Methods. Individual pixel intensity ratios were calculated for regions immediately surrounding the macropinosomes. The intensity ratios for YFP/NMTD-mCherry (gray bars) and YFP-DGKζ/NMTD-mCherry (black bars) were sorted into bins and plotted as probability distributions. The data were modeled by a three-parameter log-normal distribution (red and blue lines, respectively). Pixel ratios greater than 1 indicate the protein is more concentrated than the mCherry-NMTD membrane marker, while values less than 1 indicate a lower concentration. Asterisks indicate a significant difference in the percentages of pixels in each bin with the indicated intensity ratio.

After normalizing the signal intensities and compensating for background fluorescence (see Materials and Methods), individual pixel intensity ratios were calculated for regions immediately surrounding macropinosomes in deconvolved optical slice images. The intensity ratios for YFP and YFP-DGKζ were sorted into bins and plotted as probability distributions, which could be modeled by a three parameter log-normal distribution (Fig 5). Pixel ratios greater than 1 indicate the protein is more concentrated than the mCherry-NMTD membrane marker, while values less than 1 indicate a lower concentration. The YFP-DGKζ data was skewed to more positive values than the YFP data, suggesting DGKζ is more concentrated at and around the macropinosome membrane than YFP alone. Consistent with this, the percentage of pixels in each of the bins above an average ratio of 1.1 was significantly higher for YFP-DGKζ than for YFP alone (Fig 5). Taken together, these results suggest DGKζ is specifically concentrated at the macropinosome membrane.

### A phosphomimetic DGKζ mutant potentiates Rac1-induced macropinocytosis

**Fig 6:**
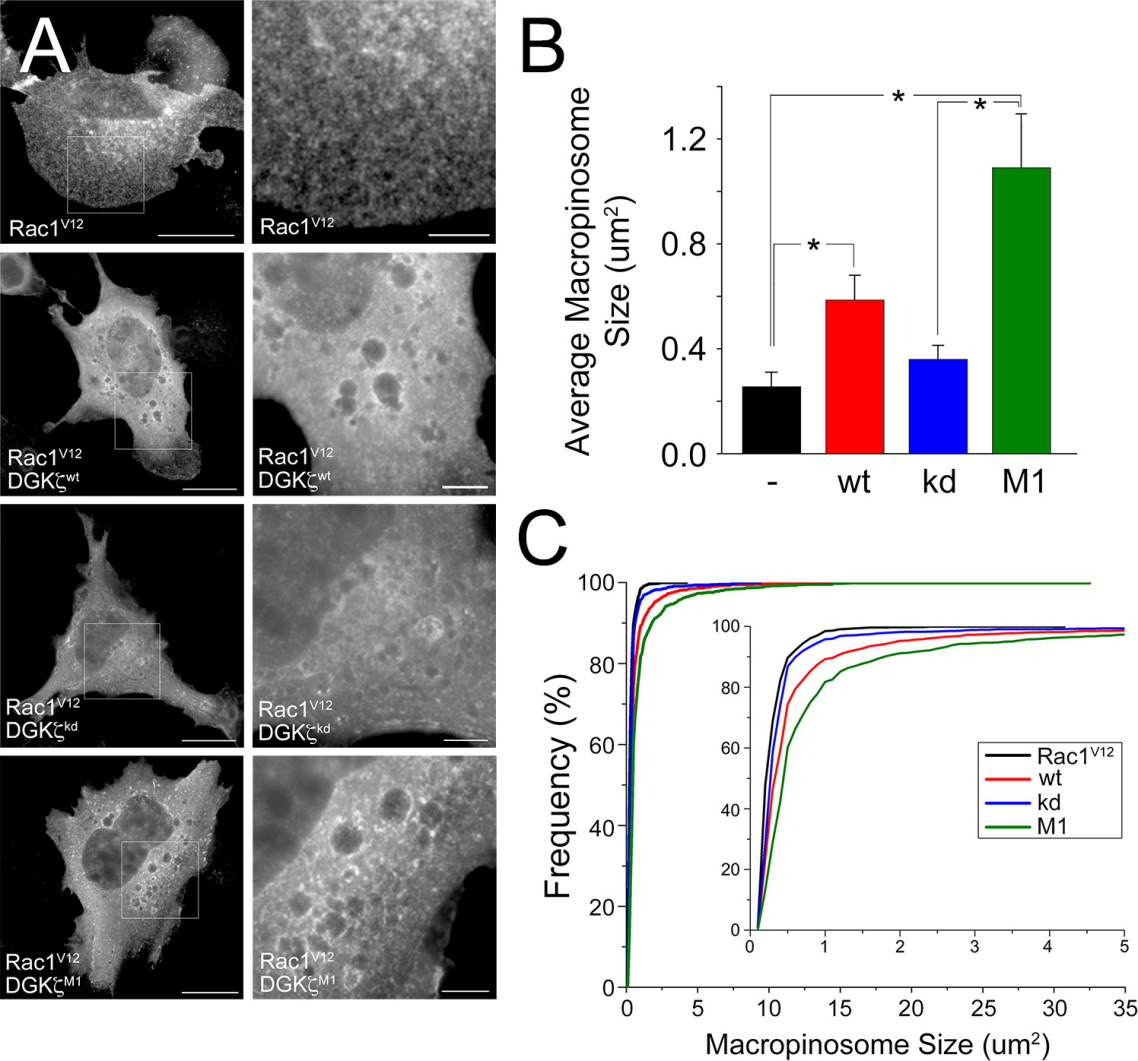
Quantitative analysis of macropinosome size in fibroblasts co-expressing Rac1^V12^ and DGKζ constructs. (A) Representative images of wild type MEFs transfected with myc-Rac1^V12^ alone or cotransfected with the indicated HA-tagged DGKζ construct. Scale bars = 20 um. Magnified images of the boxed regions are shown at the right. Scale bars = 5 um. (B) Graph showing the average macropinosome size in wild type MEFs expressing Rac1^V12^ alone (-) or Rac1^V12^ and the indicated DGKζ constructs (wt, kd or M1). Values are the mean ± SEM from three independent experiments. Statistical analysis was performed by a one-way ANOVA followed by a Tukey post-hoc multiple comparison test. Asterisks denote a significant difference (p<0.05) between the indicated conditions. (C) Graph showing the cumulative frequency distribution of macropinosome size (um^2^). The inset shows the distribution of macropinosomes between 0 and 5 um^2^.

A constitutively active Rac1 mutant (Rac1^V12^) is sufficient to promote macropinocytosis [16,20]. We previously showed DGKζ forms a multiprotein signaling complex with Rac1 and PAK1 to regulate Rac1 activation and membrane ruffling [25]. To further investigate the relationship of DGKζ and Rac1 to macropinocytosis, HA-tagged DGKζ was co-expressed with myc-tagged Rac1^V12^ in wild type MEFs. As expected, when myc-Rac1^V12^ was expressed alone in these cells it induced the formation of macropinosomes (Fig 6A). Quantification of static images of myc-Rac1^V12^-expressing cells revealed the macropinosomes had a mean size of 0.3 ± 0.05 um^2^ (Fig 6B). A cumulative frequency distribution shows that 99% of the macropinosomes had areas smaller than ~1 um^2^ (Fig 6C). When expressed alone in MEFs, none of the DGKζ constructs we tested induced membrane ruffling or macropinocytosis (not shown), consistent with our previous studies [28]. However, when wild type DGKζ was co-expressed with Rac1^V12^ (Fig 6A) there was a significant increase in the average macropinosome size to ~0.6 ± 0.09 um^2^ (Fig 6B) and an increased frequency of larger macropinosomes (Fig 6C). Co-expression of Rac1^V12^ with the MARCKS domain phosphomimetic mutant (DGKζ^M1^) led to a further increase in macropinosome size (Fig 6A–6C). In contrast, co-expression with the catalytically inactive mutant (kd) produced macropinosomes of a similar size to those produced by Rac1^V12^ alone (Fig 6A–6C). To extend the generality of these findings, we repeated these experiments in C2C12 myoblasts and obtained similar results, namely that DGKζ^M1^ significantly increased macropinosome size (S1 Fig). Western blotting confirmed that all DGKζ constructs were expressed at equivalent levels (not shown). Collectively, these results suggest DGKζ has a role in determining macropinosome size that may depend on phosphorylation of the DGKζ MARCKS domain.

## Discussion

We showed previously that DGKζ-null cells have defects in both peripheral and circular membrane ruffling [25]. In this report, we provide evidence that DGKζ has a role in growth factor-induced macropinocytosis and macropinocytosis-dependent vaccinia virus infection–both were attenuated in DGKζ-null fibroblasts.

The formation of macropinosomes from membrane ruffles is a continuous process but can be divided into a series of distinct morphological stages to facilitate quantitative analysis: (1) formation of an irregular ruffle, (2) transition to a curved (C-shaped) ruffle, (3) closure into a circular ruffle or membrane cup, and (4) cup closure, which marks the separation of the macropinosome from the plasma membrane [34]. Once closed, macropinosomes are dynamic and often extend tubules and merge with other macropinosomes (unpublished observations). This is followed by the motile phase, when a fully formed macropinosome migrates towards the nucleus. By following the fate of pre-existing membrane ruffles using time-lapse imaging and quantifying the number of ruffles that closed to form macropinosomes, we were able to establish that DGKζ-null cells have a defect in macropinosome biogenesis in addition to defective membrane ruffling.

In live imaging studies, DGKζ was associated with irregular and curved membrane ruffles and continued to be associated with membrane cups and fully formed macropinosomes. While Rac1 localization to macropinosomes was shown to be only slightly greater than on other regions of the plasma membrane, it is selectively activated at these sites after ruffle closure [34]. A recent study found that the product of DGK activity (i.e. PA) was necessary for Rac1 activation and macropinosome formation in macrophages and immature dendritic cells [38]. Our central finding (i.e. DGKζ loss reduces the frequency and size of macropinosomes) suggests DGKζ provides the PA needed for Rac1 activation during macropinocytosis. In support of this conclusion, the macropinocytosis defect in DGKζ-null fibroblasts was rescued by reintroducing catalytically active DGKζ, but not a kinase-dead mutant. It is possible also that other DGK isoforms might contribute to constitutive and growth factor-induced macropinocytosis in different cell types. Indeed, similar to DGKζ, at least one other DGK isoform, DGKα, mediates growth factor-induced membrane ruffling and Rac1 activation in hepatocytes by regulating Rac1 release from RhoGDI [39,40].

Enlarged macropinosomes caused by expression of the MARCKS domain phosphomimetic mutant DGKζ^M1^ are reminiscent of those formed by a constitutively active Rab5 mutant, which induces the formation of unusually large endosomes by homotypic fusion of early endosomes [36]. However, our live imaging analyses show DGKζ is associated with membrane ruffles and nascent macropinosomes, which argues against endosome fusion as the main mechanism for their increased size and instead, suggests DGKζ acts at a time when ruffles close to form macropinosomes. Since DGKζ^M1^ is more membrane-associated than the wild type protein [28,37], but only has half the enzymatic activity [41], it might remain associated with the macropinosome membrane longer and delay ruffle closure, giving rise to larger macropinosomes. Alternatively, DGKζ^M1^ may slow macropinosome shrinkage or the rate at which they are metabolized. Additional experiments will be required to distinguish among these possibilities.

Diacylglycerol and its non-hydrolyzable analogue phorbol myristate acetate (PMA), potently stimulate membrane ruffling and macropinocytosis [40,41]. Moreover, both DAG and PA, the product of the DGK reaction, accumulate in structures morphologically analogous to macropinocytic cups [12,42]. Exactly how these lipids drive macropinocytosis remains unclear; however, the results presented here provide a plausible mechanistic explanation for how lipid signals can be translated into cytoskeletal changes underlying macropinosome biogenesis. DAG and PMA are potent activators of protein kinase Cα (PKCα), which forms a regulated signaling complex with DGKζ[43]. PKCα-mediated phosphorylation of the MARCKS domain dissociates the complex and leads to increased association of DGKζ with the plasma membrane [28,37], where it can access DAG and metabolize it to yield PA. DGKζ-derived PA activates PAK1, which subsequently phosphorylates RhoGDI, leading to the release and activation of Rac1. Since Rac1 activity normally increases around the time of ruffle closure [34] and the PAK1 effector CtBP1/BARS has a role in macropinosome scission [21], it is likely that DGKζ also functions during these periods. Both Rac1 and PAK1 have previously been shown to play an essential role in macropinosome formation [16,20]. Therefore, it is likely that the loss of DGKζ has significant consequences for downstream signaling events that mediate the formation of macropinosomes from ruffles. Consistent with our previous results showing DGKζ directly interacts with both active and inactive Rac1 [29], the findings presented here argue that DGKζ and Rac1 function synergistically to regulate the formation and size of macropinosomes upstream and downstream of Rac1 activation.

The decreased uptake of VV particles in DGKζ-null cells (Fig 3) supports the idea that macropinocytosis is deficient in the absence of DGKζ. The large size of VV mature virions necessitates the use of macropinocytosis for cellular entry and infection because pathogens of this size are generally too big for other endocytic mechanisms [32]. VV-GFP uptake was rescued by wild type DGKζ, but not by a kinase-dead mutant, again indicating that DGKζ catalytic activity is required for macropinocytosis. In addition, VV-GFP uptake was potently blocked by a specific PAK1 inhibitor, suggesting infection does not occur via an alternative mechanism such as membrane fusion [36,37]. Since VV uptake was reduced in the absence of DGKζ, manipulating or interfering with DGKζ function might lead to novel strategies that reduce or inhibit infection of viruses and bacteria that require macropinocytosis to gain entry into cells. Clinically, modified vaccinia viruses have been adapted for use in oncolytic virus therapy [38]. Our recent finding that increased DGKζ levels and Rac1 activity can contribute to the metastatic phenotype of certain colorectal, prostate, and breast cancers [39] raises the intriguing possibility that such tumors might be more sensitive to VV uptake than neighboring normal cells and thereby potentiate oncolytic lysis. Future studies will provide additional insights into precisely how DGKζ transduces lipid signals to regulate membrane ruffling/macropinocytosis and the impact this might have on pathogenic infection.

## Materials and Methods

### Antibodies and reagents

Monoclonal hemagglutinin (HA) antibody, rat platelet-derived growth factor-BB, cell culture grade glutamine and penicillin-streptomycin were all from Sigma-Aldrich (St. Louis, MO). Monoclonal c-myc (9E10) antibody was from Roche Applied Science (Indianapolis, IN). FuGENE 6 transfection reagent was from Roche Diagnostics (Indianapolis, IN). AlexaFluor 488- and 594-conjugated secondary antibodies, AlexaFluor 594- conjugated phalloidin, and Texas-Red dextran (70 kDa) were from Invitrogen (Carlsbad, CA). Horseradish peroxidase-conjugated anti-rabbit and anti-mouse secondary antibodies were from Jackson ImmunoResearch Laboratories, Inc. (West Grove, PA). Matrigel was from BD Biosciences.

### Plasmids and constructs

Plasmids encoding wild-type (wt) DGKζ, a membrane-associated mutant mimicking phosphorylation of the MARCKS domain (DGKζ^M1^), and a kinase-dead mutant (DGKζ^kd^), all with three tandem, N-terminal HA epitope tags, have been previously described (Topham et al, 1998; Hogan et al, 2001). N-terminal myc-tagged Rac1^V12^ in pEFmPLINK was a gift from Dr. Andrew Thorburn (University of Colorado, Denver, CO). N-terminal yellow fluorescent protein (YFP)-tagged Rac1^V12^ has been previously described (Abramovici, 2009). CFP-DGKζ^M1^ was generated by subcloning DGKζ^M1^ from pcDNA3.1 into pECFP-C1 (Clonetech) using EcoRI and XbaI restriction sites. Cloning and production of adenoviral constructs encoding green fluorescent protein (GFP), DGKζ^WT^, DGKζ^M1^, or DGKζ^kd^ have been described previously (Yakubchyk et al., 2005). A plasmid encoding YFP-DGKζ was made by first subcloning DGKζ in pCMV-HA [28] into pcDNA3.1 using EcoR I and Not I restriction sites. Then, the resulting plasmid was digested with EcoR I and Xba I enzymes and ligated into pEYFP-C1 (Clonetch, Mountain View, CA) digested with the same enzymes.

### Cell culture, transfection/infection and immunofluorescence microscopy

Wild type and DGKζ-deficient immortalized mouse embryonic fibroblasts (MEFs) were generated as previously described (Abramovici, 2009). All animal experimental studies were approved by the University of Ottawa Animal Care Committee and conformed to the guidelines set forth by the Canadian Council on Animal Care and Canadian Institutes of Health Research. MEFs were cultured under standard growth conditions in Dulbecco’s Modified Eagle Medium (DMEM) supplemented with 10% fetal calf serum, 2 mM L-glutamine, 100 U ml^–1^ penicillin, and 100 U ml^–1^ streptomycin and grown at 37°C in a humidified incubator with 5% CO_2_. C2C12 myoblasts were maintained as described previously [42]. Fibroblasts were seeded onto Matrigel-coated coverslips in 24-well dishes at a density of 40,000 cells per well, serum-starved overnight then stimulated with either PDGF or vehicle (0.14M HCL and 0.1% bovine serum albumin). Cells were fixed and processed as described previously (Abramovici, 2009). For experiments using Rac1^V12^, cells were transfected with myc- or YFP-tagged Rac1^V12^ constructs for 24 h. In some experiments, HA- or YFP-tagged DGKζ, DGKζ^M1^, or DGKζ^kd^ constructs were cotransfected with myc-tagged Rac1^V12^. Cells were visualized by time-lapse microscopy or were fixed in 4% paraformaldehyde and processed for immunocytochemistry. For rescue experiments, DGKζ-null fibroblasts were infected with adenoviruses encoding DGKζ constructs at a multiplicity of infection of 100 for 1 h at 37°C. Cells were incubated a further 24 h under standard growth conditions as described previously [25].

### Phase contrast live imaging

Wild-type and DGKζ knockout fibroblasts were seeded on 35mm culture dishes (MatTek, Ashland, MA) and grown to 70–80% confluence. The cells were serum-starved overnight in serum-free DMEM containing penicillin/streptomycin and glutamine. The next day, the cells were washed twice in pre-warmed, serum-free DMEM then placed in a stage-fixed cell chamber at 5% CO_2_, 37°C prior to imaging. The cells were stimulated with 25 ng/mL platelet-derived growth factor (PDGF) then filmed for 30 minutes using an Axiovert 200M microscope (Carl Zeiss, Germany) and EC plan Neofluar 40x/1.30 oil objective (for high-resolution images) or 10x objective (for quantitation of macropinosome size and number). Phase-contrast images were captured every 10 sec using an Axiocam HRm charged-coupled device camera. With phase-contrast light microscopy macropinosomes are readily observed as large, phase-bright vesicles [43]. Images were recorded using AxioVision software (version 4.6), then exported and processed using Adobe Photoshop.

### Macropinosome quantification from time lapse images

Macropinosomes were defined as circular, phase-bright organelles that dissociated from circular ruffles or peripheral ruffles in cells stimulated with PDGF. For each cell in a field, successful transitions from membrane ruffles to macropinosomes were quantified. To calculate the percentage of cells with macropinosomes, the number of cells with at least one macropinosome was divided by the total number of cells in the field. To calculate the number of macropinosomes per cell, the total number of observed macropinosomes was divided by the number of cells containing at least one macropinosome. To quantify macropinosome surface area in fibroblasts, cells were imaged at 10x magnification, and the macropinosomes were traced using the “outline” tool in AxioVision. Data were graphed using SigmaPlot 12.5.

To quantify the number and area of Rac1^V12^-induced vesicles in fibroblasts or C2 myoblasts, the transfected cells were fixed and stained with monoclonal antibody 9E10 against myc epitope-tagged Rac1. The cells were photographed on a Zeiss Axioscop 2 microscope fitted with a 40x objective using a Zeiss Axiocam digital camera. The captured images were processed using the AxioVision software as follows: Vesicles were outlined in white using the circle/ellipse tool. The annotated images were then saved in the (8 bit) tagged image file format (TIFF). The saved images were imported into Image J. First, the Set Scale function was used to convert pixels into a known distance with the calibration settings obtained from the microscope. Then the images were processed using the Thresholding tool until only the annotated vesicle outlines were visible. The analyze particle function was used to determine the number and area of the vesicles.

### Vaccinia virus uptake

Equivalent numbers or wild type or DGKζ-null fibroblasts plated on plastic dishes in serum-free medium were infected with equal volumes (multiplicity of infection = 1) of a Vaccinia virus strain engineered to express GFP in the cytoplasm of infected cells McCart, 2001. The cells were incubated in a humidified chamber at 37°C with 5% CO_2_ for 6 h to allow for GFP expression and then were lysed and extracted as described (Ard et al., 2012). Equivalent amounts of protein were analyzed by SDS-PAGE and immunoblotting with an anti-GFP antibody, followed by a horseradish peroxidase-conjugated secondary antibody. Bound antibody was detected by chemiluminescence. Digital images of western blots were captured using a Kodak Image Station 440 CF (Rochester, NY). The band intensities were measured by densitometric analysis and normalized to the relative amount of tubulin in each sample. For rescue experiments, DGKζ-null cells were infected with infected with adenovirus bearing HA-tagged DGKζ^wt^, DGKζ^M1^, or DGKζ^kd^ as described previously (Ard et al., 2012) and were incubated at 37°C with 5% CO_2_ for 36 hours to allow for protein expression before infecting with VV.

### Fluorescence imaging and quantification

Live cell imaging experiments were carried out using a wide-field deconvolution-based fluorescence microscope system (DeltaVision CORE; Applied Precision, Issaquah, WA) equipped with a three-dimensional motorized stage, temperature-and gas-controlled environmental chamber, Xenon light source, and quantifiable laser module. Images were collected using a 60 × NA 1.4 Plan-Apochromat objective and recorded with a CoolSNAP coupled-charge device (CCD) camera (Roper Scientific, Trenton, NJ). The microscope was controlled by SoftWorX acquisition and deconvolution software (Applied Precision, Seattle, WA). MEFs were seeded at 15% confluence onto optically clear 35 mm dishes (ibidi, Verona, WI) then incubated overnight in a humidified chamber at 37°C with 5% CO_2_. The cells were cotransfected with pTK165_Mem-mCherry-4.1G-CTD (Addgene Plasmid 46362 from Dr. Iain Cheeseman) encoding the N-terminal palmitoylation domain (20 amino acids) of neuromodulin (GAP-43) fused to the N-terminus of mCherry (NMTD-mCherry), and either YFP-DGKζ or an empty YFP expression vector using Effectene transfection reagent (Qiagen Inc., Toronto, ON). Eight hours following transfection, the cells were washed with phosphate buffered saline, pH 7.4 and the media was replaced with phenol- and serum-free medium and incubated for an additional 16 hours.

On the day of imaging, the media was replaced with phenol- and serum-free media supplemented with 25 mM HEPES, pH 7.4. The cells were placed into the microscope environmental chamber and allowed to acclimatize for 30 minutes before stimulating with 50 ηg/ml PDGF-BB (Sigma-Aldrich, St. Louis, MO). Imaging commenced four minutes after stimulation to allow for re-centering of cotransfected cells within the field of view at each location. For each cell, a stack of five (512 x 512 pixels, 4.673 pixels/μm) images with 0.75μm separation was acquired in the using the Sedat quad filter set: FITC/GFP (485 ± 20 nm Excitation, 535 ± 30 Emission) and TRITC (560 ± 25 nm Excitation, 607 ± 36 nm Emission (Chroma Technology Corp, Bellows Falls, VT). Images were captured every 15–20 seconds for approximately 20 minutes. Typical exposures times were 0.25–0.4 seconds.

Deconvolution of the images was performed using the SoftWorX software and the files were imported into Image J software (version 1.47f) using the Bio-Formats plug-in Linkert, 2010. Variation in the expression of the tagged constructs was compensated for by splitting the colour channels of each image set into separate files and normalizing each using the enhance contrast option with 0.1% saturation. The divide operation of the image calculator tool set was then used to produce an image where each pixel represents the ratio of YFP to mCherry signal.

To measure YFP-DGKζ fluorescence on macropinosome membranes, the perimeter of individual macropinosomes was selected using the Freehand Selection Tool in Image J. The interior of the macropinosome was subtracted from each selection then the intensity of each pixel within the selection area was recorded in a text file using a macro written with Image J. To subtract the background fluorescence, the pixel intensity values were normalized to the average of the mean gray values from three separate regions surrounding each macropinosome.

## Supporting Information

S1 Fig Quantitative analysis of macropinosome size in C2C12 myoblasts co-expressing Rac1^V12^ and DGKζ constructs. (A—C) Representative images of C2 myoblasts transfected with myc-tagged Rac1^V12^ alone or cotransfected with the indicated HA-tagged DGKζ constructs. Scale bars, 20 um. (D–F) Quantification of vesicle size. Vesicle areas were quantified as described in Materials and Methods and the resulting values were sorted into bins and plotted as histograms. The graphs show the percentage of vesicles as a function of their size (in um^2^). The axes in (F) are the same for (D) and (E).
(TIF)

S1 Movie Time-lapse observations of wild type MEFs stimulated with PDGF. Wild type fibroblasts were serum-starved for 24 h then stimulated with 50 ηg/ml PDGF and immediately visualized by time-lapse, phase contrast microscopy. Images were acquired at 10 s intervals over a period of 30 min. The video is displayed at 18 frames/second. Scale bar = 10 um.
(AVI)

S2 Movie Higher magnification view of macropinosome formation in wild type MEFs. Images were acquired at 10 s intervals over a period of 30 min. The video is displayed at 18 frames/second. Scale bar = 10 um.
(AVI)

S3 Movie Time-lapse observation of DGKζ-null MEFs stimulated with PDGF. DGKζ-null fibroblasts were serum-starved for 24 hr then stimulated with 50 ηg/ml PDGF and immediately visualized by time-lapse, phase contrast microscopy. Images were acquired at 10 s intervals over a period of 30 min. The video is displayed at 18 frames/second. Scale bar, 10 um.
(AVI)

S4 Movie Time-lapse observation of a wild type MEF expressing YFP-DGKζ and NMTD-mCherry. Wild type fibroblasts were transiently transfected with YFP-DGKζ (green) and NMTD-mCherry (red) and were visualized 24 h later by time-lapse fluorescence microscopy after stimulation with 50 ηg/ml PDGF-BB. One of five deconvolved optical sections is shown. Scale bar = 10 um.
(AVI)

S5 Movie Time-lapse observation of a wild type MEF expressing YFP and NMTD-mCherry. Wild type fibroblasts were transiently transfected with YFP (green) and NMTD-mCherry (red) and were visualized 24 h later by time-lapse fluorescence microscopy after stimulation with 50 ηg/ml PDGF-BB. One of five deconvolved optical sections is shown. Scale bar = 10 um.
(AVI)

## Abbreviations

DAG: diacylglycerol
DGK: diacylglycerol kinase
DMEM: Dulbecco’s Modified Eagle Medium
HA: hemagglutinin
HRP: horseradish peroxidase
PA: phosphatidic acid
PAK1: p21-activated kinase 1
PDGF: platelet-derived growth factor
YFP: yellow fluorescent protein
