# Supplementary figures and images for: Regulation of Macropinocytosis by Diacylglycerol Kinase ζ
Source: PLoS One. 2015 Dec 23;10(12):e0144942. doi: 10.1371/journal.pone.0144942 (PMC4689489; doi:10.1371/journal.pone.0144942)

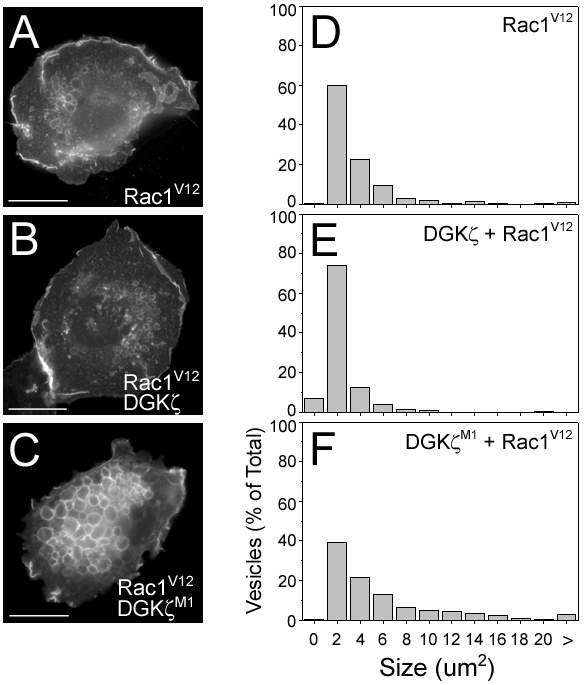

Supplement: S1 Fig — (A—C) Representative images of C2 myoblasts transfected with myc-tagged Rac1V12 alone or cotransfected with the indicated HA-tagged DGKζ constructs. Scale bars, 20 um. (D–F) Quantification of vesicle size. Vesicle areas were quantified as described in Materials and Methods and the resulting values were sorted into bins and plotted as histograms. The graphs show the percentage of vesicles as a function of their size (in um2). The axes in (F) are the same for (D) and (E). (TIF) [file pone.0144942.s001.tif]
